# WHSC1 is involved in DNA damage, cellular senescence and immune response in hepatocellular carcinoma progression

**DOI:** 10.1111/jcmm.17743

**Published:** 2023-04-18

**Authors:** Jia Yan, Ming Yang Zhang, Jing Lin, Ke Xin Li, Zhi Min Zhao, Yu Min Gao, Xiu Ling Deng, Chang Shan Wang, Hai Sheng Wang

**Affiliations:** ^1^ School of Basic medical Inner Mongolia Medical University Hohhot Inner Mongolia China; ^2^ School of Life science Inner Mongolia University Hohhot Inner Mongolia China; ^3^ Department of Nutrition Affiliated Hospital of Inner Mongolia Medical University Hohhot Inner Mongolia China; ^4^ School of Public health Inner Mongolia Medical University Hohhot Inner Mongolia China

## Abstract

Wolf–Hirschhorn syndrome candidate 1 (WHSC1) is a transcriptional regulatory protein that encodes a histone methyltransferase to control H3K36me2 modification. WHSC1 was upregulated and associated with poor prognosis in HCC. The elevated WHSC1 likely due to the alterations of DNA methylation or RNA modification. WHSC1 perhaps form a chromatin cross talk with H3K27me3 and DNA methylation to regulate transcription factors expression in HCC. Functional analysis indicated that WHSC1 was involved in DNA damage repair, cell cycle, cellular senescence and immune regulations. Furthermore, WHSC1 was associated with the infiltrating levels of B cell, CD4+, Tregs and macrophage cells. Therefore, our findings suggested that WHSC1 might function as a promotor regulator to affect the development and progression of HCC. Thus, WHSC1 could be a potential biomarker in predicting the prognosis and therapeutic target for patients with HCC.

## 1 INTRODUCTION

HCC is one of the most common primary malignant liver tumours with high incidence and mortality. Although the targeted therapy and immunotherapy have achieved encouraging results, it still had a poor overall survival rate in patients with advanced HCC. Therefore, more reliable prognostic markers and novel regulatory mechanisms identification is crucial for to improve the survival rate of HCC.

DDR pathway has become the target of antitumour drugs.^1^ It has been reported that DDR is involved in prognosis, tumour initiation, metastasis and antitumor immune responses.^2^, ^3^ DDR status has been confirmed to serve as a potential biomarker to predict opposite clinical prognosis of immunotherapy. WHSC1 encodes a SET domain‐containing histone methyltransferase, which could catalyse the di‐methylation of H3K36 to involve in gene transcriptional regulation.^4^ However, further study is necessary for the function and carcinogenic mechanism of WHSC1 in HCC. To fully assess the role of WHSC1 in HCC, we comprehensively investigated the gene expression, prognosis, epigenetic regulation and functions of WHSC1 in HCC.

## 2 MATERIALS AND METHODS

### 2.1 Gene expression and survival analysis

The mRNA and protein level of WHSC1 was confirmed based on UALCAN‐LIHC and HPA data.^5^ Kaplan–Meier survival analysis was used to assess the prognosis of WHSC1 in HCC.

### 2.2 The functional enrichment analysis of WHSC1

The GSEA was used to evaluate the KEGG and GO enrichment of WHSC1. The proteins interaction networks of WHSC1‐related genes were constructed using the Cytoscape base on the STRING data.^6^

### 2.3 Cell function assays

The assay was performed in accordance with the manufacturer's protocol. The proliferation of HCC cell was evaluated using the CCK‐8 assay (Mei5bio, China). The cell cycle was detected using flow cytometer.

### 2.4 Immune infiltration analysis

The TIMER, CAMOIP and GSCA were used to calculate the abundance of tumour infiltrating immune cells in tumour tissues of LIHC.^7^

### 2.5 Statistical analysis

All the experiment was independently repeated three times. Data were summarized as the mean ± SD. The results were considered to be statistically significant when the value of *p* was <0.05.

## 3 RESULTS

### 3.1 Relationships between the clinicopathological and prognostic features of WHSC1 in HCC

**FIGURE 1:**
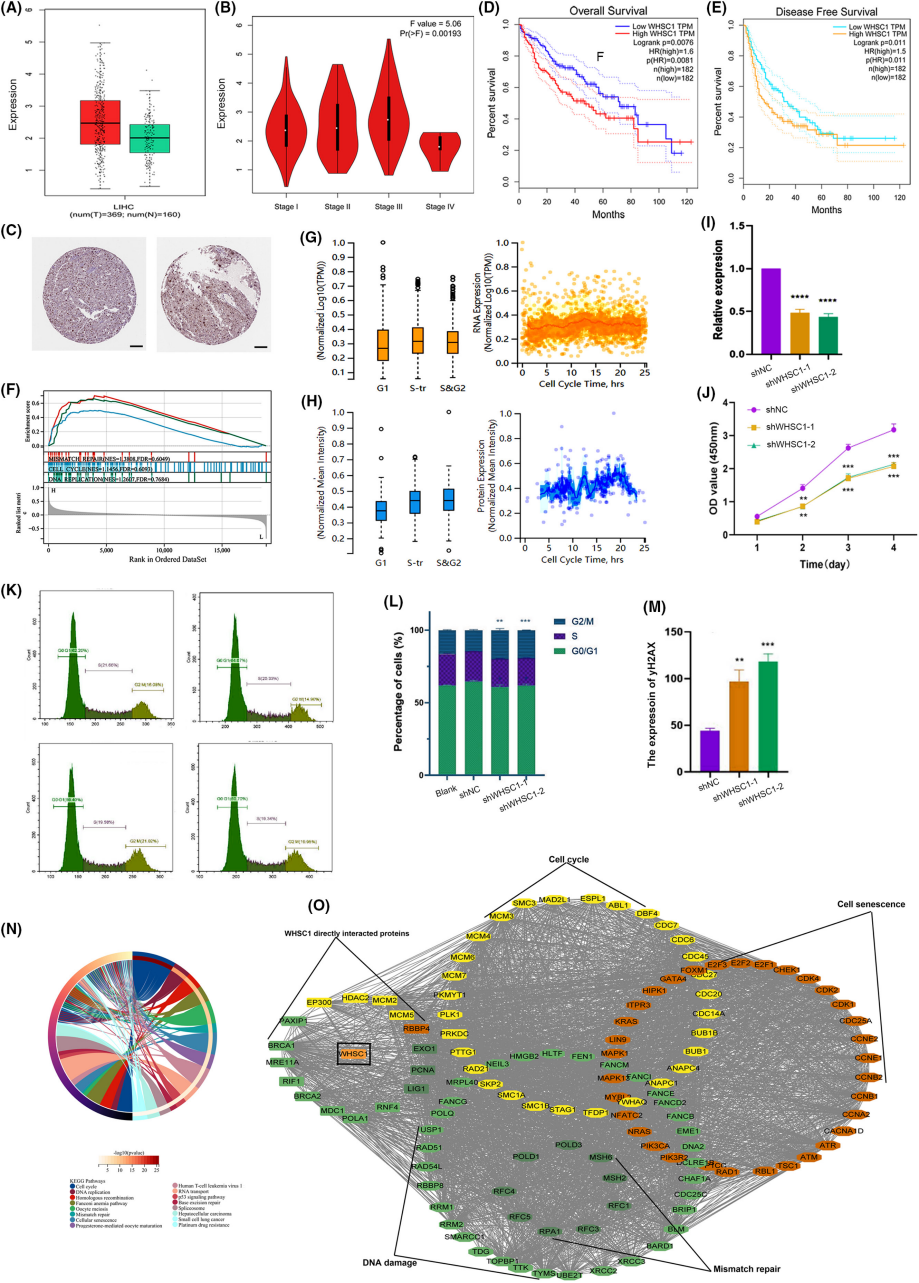
Gene expression, prognosis and function of WHSC1. (A) The mRNA level of WHSC1 in LIHC. (B) The mRNA level of WHSC1 in different stage of patients with LIHC. (C) The protein expression of WHSC1 in patients with LIHC, that was showed by immunohistochemical images. Scale bar, 100 μM. (D, E) Correlation between WHSC1 gene and the survival outcome in LIHC. The Kaplan–Meier curves with positive results are used to display the OS (D) and DFS (E). (F) WHSC1 was associated with mismatch repair, cell cycle and DNA replication based on the GSEA enrichment analysis. (G, H) The mRNA (G) and protein (H) expression level of WHSC1 in cell cycle processes. (I, J) The down‐regulated WHSC1 inhibits the ability of HCC cell proliferation. (K, L) The down‐regulated WHSC1 results in cell cycle arrest. (M) The expression of yH2AX was increased after down‐regulated WHSC1 in HCC cell. (N) The KEGG enrichment analysis of WHSC1‐related genes in LIHC. (O) The protein interaction network of WHSC1 associated genes in DNA damage, mismatch repair, cell cycle and cellular senescence pathways. *p < 0.05, **p < 0.01, ***p < 0.001, ****p < 0.0001. The *p* < 0.05 is considered statistically significant.

In order to evaluate the role of WHSC1 in cancers, we assessed the mRNA level of WHSC1 and found it showed lowest level in liver tissues (Figure S1). However, it was abnormally upregulated in tumour tissues with the highest level at the stage III of HCC (Figure 1A,B). The immunohistochemistry results indicated that protein level of WHSC1 was also increased (Figure 1C), and the elevated WHSC1 was significantly associated with poor prognosis in HCC (Figure 1D,E). Furthermore, WHSC1 mRNA was positively correlated with most of m6A methylation regulatory factors and showed hypomethylation at promoter region, suggesting that elevated WHSC1 is attributed to alterations of DNA methylation or RNA modification. (Figure S2A–C). Moreover, the probes cg00249632 and cg18675616 showed significant association of WHSC1 methylation and worse prognosis (Figure S2D,E). Therefore, WHSC1 was an important prognostic factor in HCC.

### 3.2 WHSC1 is associated with other epigenetic modifications in HCC progression

WHSC1 coexpressed genes were enriched in PRC2 complex pathway. WHSC1 expression was significantly associated with PRC2 member, *EZH2*, *SUZ12* and *EED* (Figure S3A–D). Additionally, we also assessed the relationships between *WHSC1* and DNA methylation enzymes. WHSC1 was positively associated with *DNMT1*, *DNMT3A* and *DNMT3B* (Figure S3E–G). Furthermore, WHSC1 could directly interact with PRC2 complex and DNMTs, suggesting that WHSC1 likely associated with these epigenetic modifications to regulate genes transcription in HCC.

### 3.3 WHSC1 was involved in DNA damage, cell cycle, cellular senescence in HCC

**FIGURE 2:**
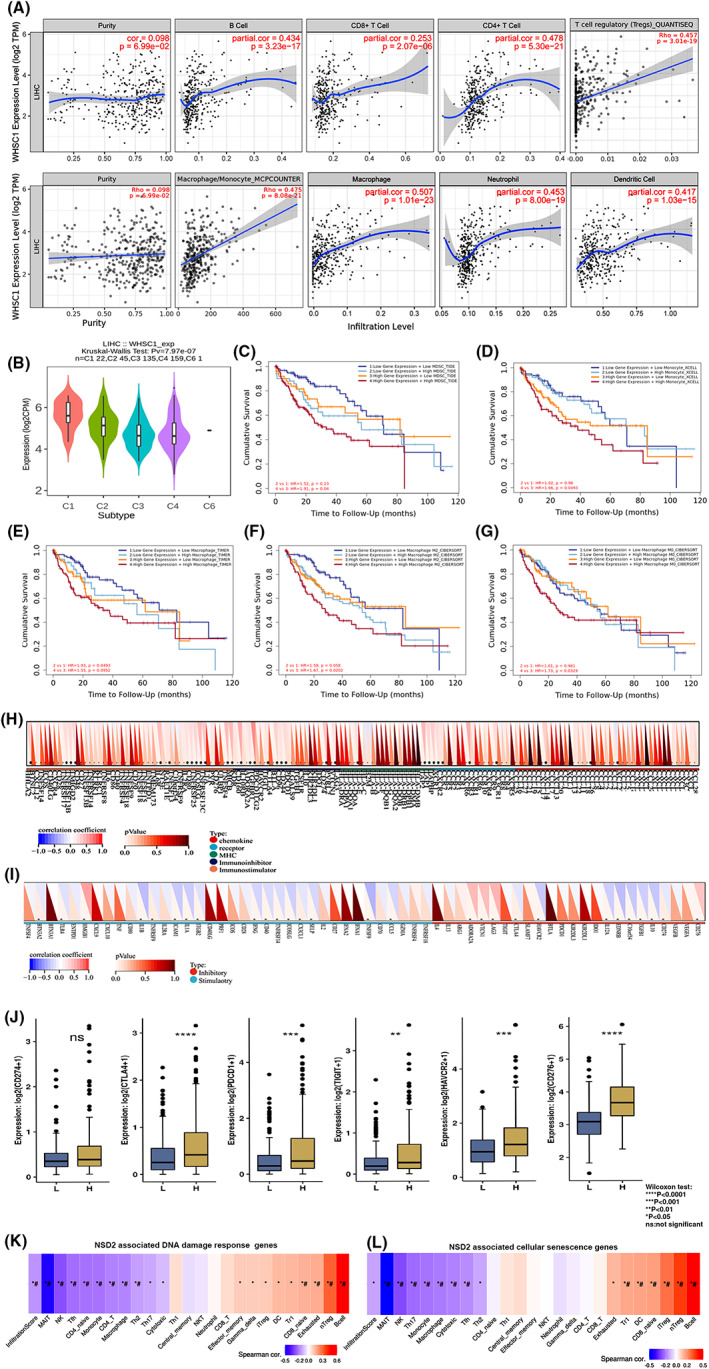
The correlation of WHSC1 expression with immune infiltration level in LIHC. (A) Correlation analysis between the expression of WHSC1 and immune infiltration of B cell, CD4 + T cell, CD8 + T cell, Tregs, tumour‐associated monocyte/macrophage, and neutrophil and dendritic cell in LIHC. (B) The relationships of WHSC1 expression and immune subtypes including wound healing (C1), inflammatory (C2), IFN‐gamma dominant (C3), and lymphocyte depleted (C4) and TGF‐b dominant (C5) immune subtypes. (C–G) The Kaplan–Meier overall survival (OS) curves of patients with LIHC based on the WHSC1 expression and immune cells infiltration levels. High level of WHSC1 expression and MDSC (C), monocyte (D), macrophage (E), M2 and M0 macrophage (F, G) showed worse prognosis in LIHC. *p < 0.05. The *p* < 0.05 is considered statistically significant. (H, I) Correlations of WHSC1 coexpressed genes with infiltration levels of different immune cell subtypes in HCC. Red represents positive correlation; blue represents negative correlation. (H, I) The relationships between WHSC1 and immune‐related genes including chemokine, receptor, MHC, immunoinhibitor, and immunostimulatory (H) or immune‐related inhibitory and stimulatory (I). (J) The correlation of WHSC1 expression and immune checkpoint‐associated genes including CD274, CTLA4, PDCD1, HAVCR2 and CD276 in LIHC. (K) WHSC1 coexpressed DNA damage response genes, (L) WHSC1 coexpressed cellular senescence genes in LIHC. *indicates *p* value ≤0.05, #represents FDR ≤0.05. *P* value <0.05 is considered statistically significant.

The functional enrichment analysis was performed based on GSEA assay. We found that WHSC1 was significantly enriched in mismatch repair, cell cycle and DNA replication pathways (Figure 1F). Furthermore, we detected the expression of WHSC1 in cell cycle. Its expression was increased in the S and G2 stage (Figure 1G,H). To confirm function of WHSC1, the shRNA was transfected to downregulate WHSC1 level in HCC. The CCK‐8 assay showed that cell proliferation and cycle was abnormal arrested (Figure 1I–L). Moreover, the yH2AX, a biomarker of DSB, was increased in down‐regulated WHSC1 HepG2 cell (Figure 2N). Therefore, WHSC1 was involved in cell cycle and DNA damage in HCC.

To further confirm role of WHSC1, we obtained all the WHSC1‐associated genes in LIHC based on RNA‐seq results. KEGG analysis indicated that these genes were involved in cell cycle, DNA replication, mismatch repair and cellular senescence (Figure 1N). Furthermore, we constructed the protein interaction network of these genes (Figure 1O). DNA damage response genes, PAXIP1, BRCA1, MRE11A, RIF1, BRCA2, MDC1, POLA1, RNF4, mismatch repair‐related genes, EXO1, PCNA and LIG1, cellular senescence‐related gene RBBP4, and cell cycle‐related genes EP300, HDAC2, MCM2 and MCM5 directly interact with WHSC1.

### 3.4 Relationship between WHSC1 and tumour immune microenvironment

We further analysed the relationships of WHSC1 and MSI or stemness. The strong correlations were found between *WHSC1* expression and MSI or stemness (Figure S4A,B). Moreover, stemness‐related genes were associated with PD‐L1 pathways. Therefore, we evaluated the function of WHSC1 in immune response. GO analysis showed that WHSC1 was mainly involved in several immune pathways (Table S1). WHSC1 expression was significantly positively associated with macrophages, monocyte, CD4 + T, Treg, neutrophil and dendritic cells infiltration (Figure 2A). It was increased in wound healing and inflammatory immune subtypes (Figure 2B). Moreover, the prognosis of higher level *WHSC1* with high immune cells infiltration, including MDSC, monocyte, macrophage was significantly worse than that in low‐infiltrating groups (Figure 2C–E). Higher level M2 and M0 macrophage with *WHSC1* expression showed a worse prognosis (Figure 2F,G). Furthermore, it's expression was associated with the expression of chemokine, receptor, MHC, immunoinhibitory or immunostimulator genes and immune checkpoint genes (Figure 2H–J). Additionally, WHSC1‐related DNA damage and cell senescence genes also associated with immune infiltration of Treg and B cells (Figure 2K,L).

## 4 DISCUSSION

It is well known that DNA damage repair pathways is correlated with variability in HCC clinical outcomes. WHSC1 specifically catalyses H3K36me2, which will result in conformation of chromatin, transcriptional regulation to control cell growth, DNA damage and apoptosis in several cancers.^4^ It has been reported that WHSC1‐mediated di‐methylation of PTEN at K349 is recognized by the tudor domain of 53BP1 to recruit PTEN to DNA damage sites and result in dephosphorylation of γH2AX for governing efficient repair of DSBs.^8^

Recently, studies have indicated that WHSC1 is involved in antitumor immunity.^9^, ^10^ The down‐regulated WHSC1 expression will diminish MHC‐I levels, impair antitumor immunity and blunt the effect of immune checkpoint blockade.^9^ WHSC1 directly interact with NLRC5 to promote MHC‐I gene expression and results in impairing the IFN‐γ‐stimulated antitumor immunity.^10^ It also acts as a critical epigenetic regulator for follicular helper T cell differentiation and Treg recruitment.^11^, ^12^

Compelling evidence has shown that DNA damage response deficiency is associated with activation of anticancer immunity. The alterations of DDR genes can serve as biomarkers for the selection of suitable patients to receive specific therapeutics.^13^ It has been reported that cGAS‐cGAMP‐STING pathway connects DNA damage to inflammation and cellular senescence.^14^ Our results indicated that WHSC1 perhaps serve as a link from DNA damage to cellular senescence and immune response. In mechanism, WHSC1 likely results in H3K36me2 modification to combined with PRC2‐related H3K27me3, or DNMTs‐related DNA methylation to affect the transcriptional regulation of DNA damage, cellular senescence or immunity‐related genes expression in HCC.

## AUTHOR CONTRIBUTIONS

**jia yan:** Conceptualization (lead); data curation (lead); visualization (lead); writing – original draft (lead); writing – review and editing (lead). **ming yang Zhang:** Data curation (lead); methodology (lead); supervision (lead). **Jing Lin:** Data curation (equal); investigation (equal); methodology (equal); resources (equal); software (equal); supervision (equal). **ke xin Li:** Data curation (equal); formal analysis (equal); resources (equal); software (equal). **zhi min Zhao:** Data curation (equal); formal analysis (equal); investigation (equal); methodology (equal); visualization (equal). **yu min Gao:** Data curation (lead); project administration (equal); supervision (equal); validation (equal). **xiu ling Deng:** Formal analysis (supporting); methodology (supporting); software (equal); supervision (supporting). **chang shan wang:** Funding acquisition (lead); project administration (lead). **Haisheng Wang:** Conceptualization (equal); funding acquisition (lead); project administration (lead).

## FUNDING INFORMATION

This work was supported by National Natural Science Foundation of China (CN) (grant Nos. 81660024). Science and Technology Innovation Guidance Project of Inner Mongolia Region (Grant Nos. KCBJ2018021). The Key Natural Science Foundation of Universities Project of Inner Mongolia (Grant Nos. NJZZ22686). Science and Technology Innovation team of Inner Mongolia Medical University (YKD2022TD031).

## CONFLICT OF INTEREST STATEMENT

The authors declared that they have no conflict of interest.

## Supporting information


Figure S1.

Figure S2.

Figure S3.

Figure S4.

Table S1.

AppendixS1.

## Data Availability

The datasets used in this article are publicly available as described in Materials and Methods. All data generated during this study are included in this published article and its supplementary information files. The data available in TCGA (https://portal.gdc.cancer.gov/) databases. The datasets analysed during the current study are available from the corresponding author on reasonable request.
