# Supplementary figures and images for: WHSC1 is involved in DNA damage, cellular senescence and immune response in hepatocellular carcinoma progression
Source: J Cell Mol Med. 2023 Apr 18;27(10):1436–41. doi: 10.1111/jcmm.17743 (PMC10183708; doi:10.1111/jcmm.17743)

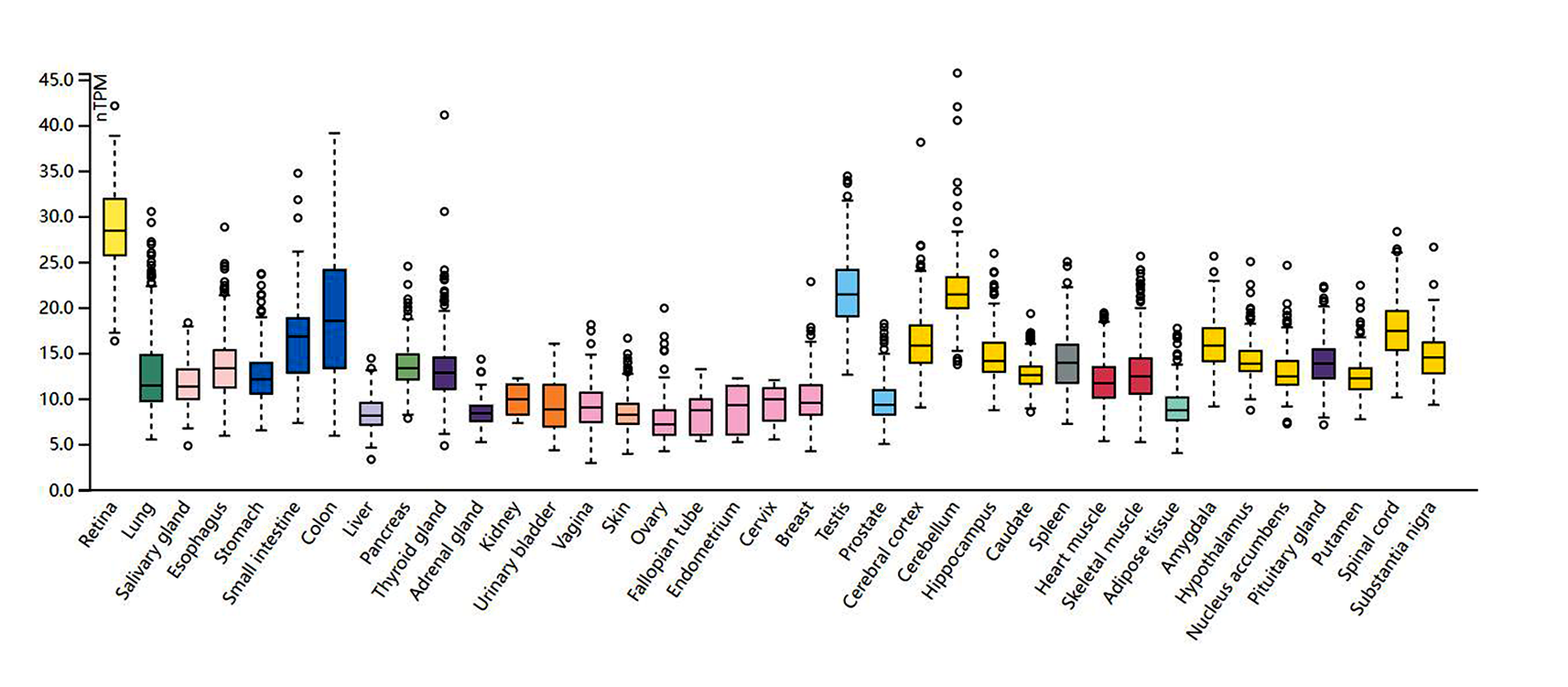

Supplement: Supplementary file 1 — Figure S1. [file JCMM-27-1436-s003.tif]

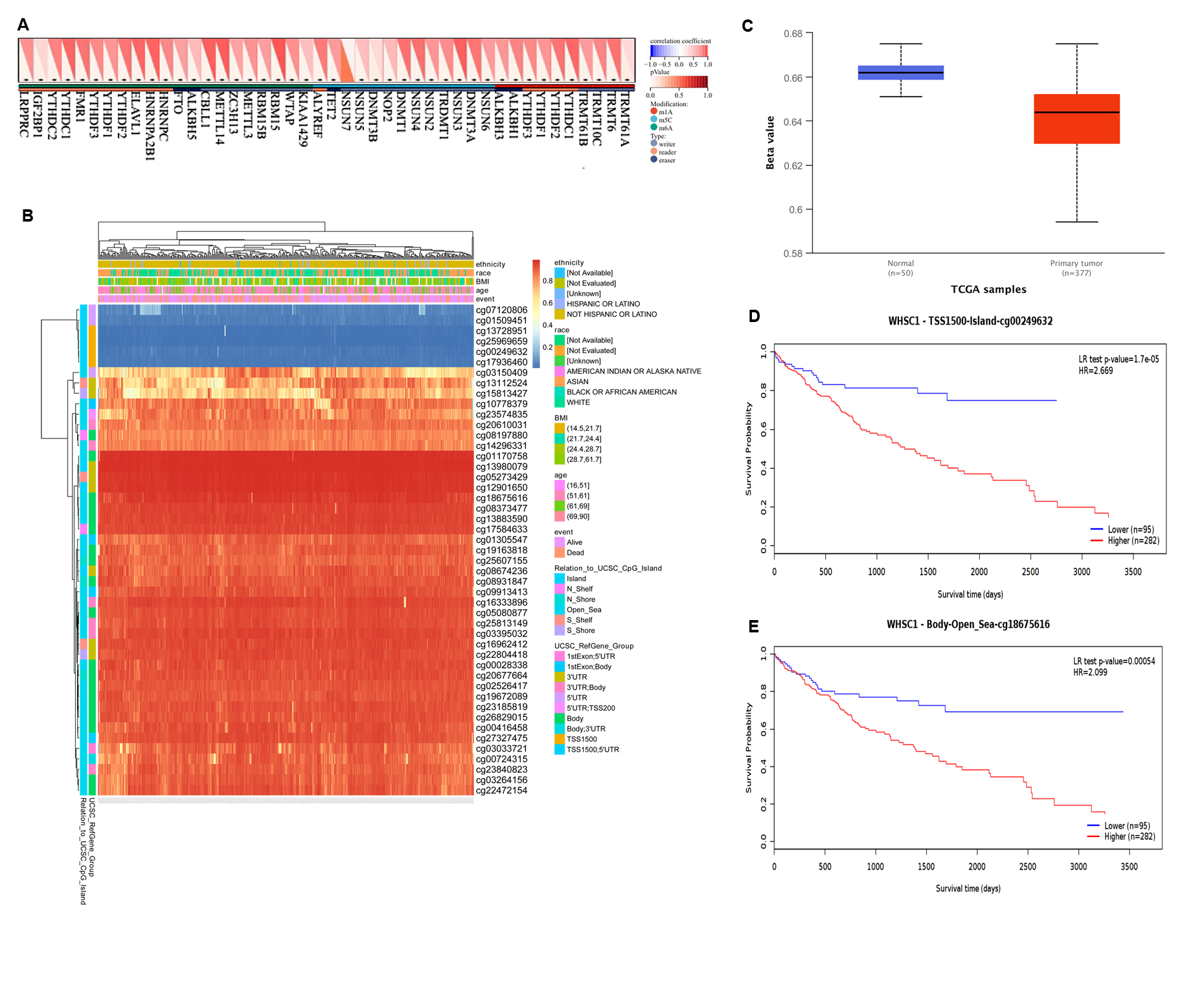

Supplement: Supplementary file 2 — Figure S2. [file JCMM-27-1436-s002.tif]

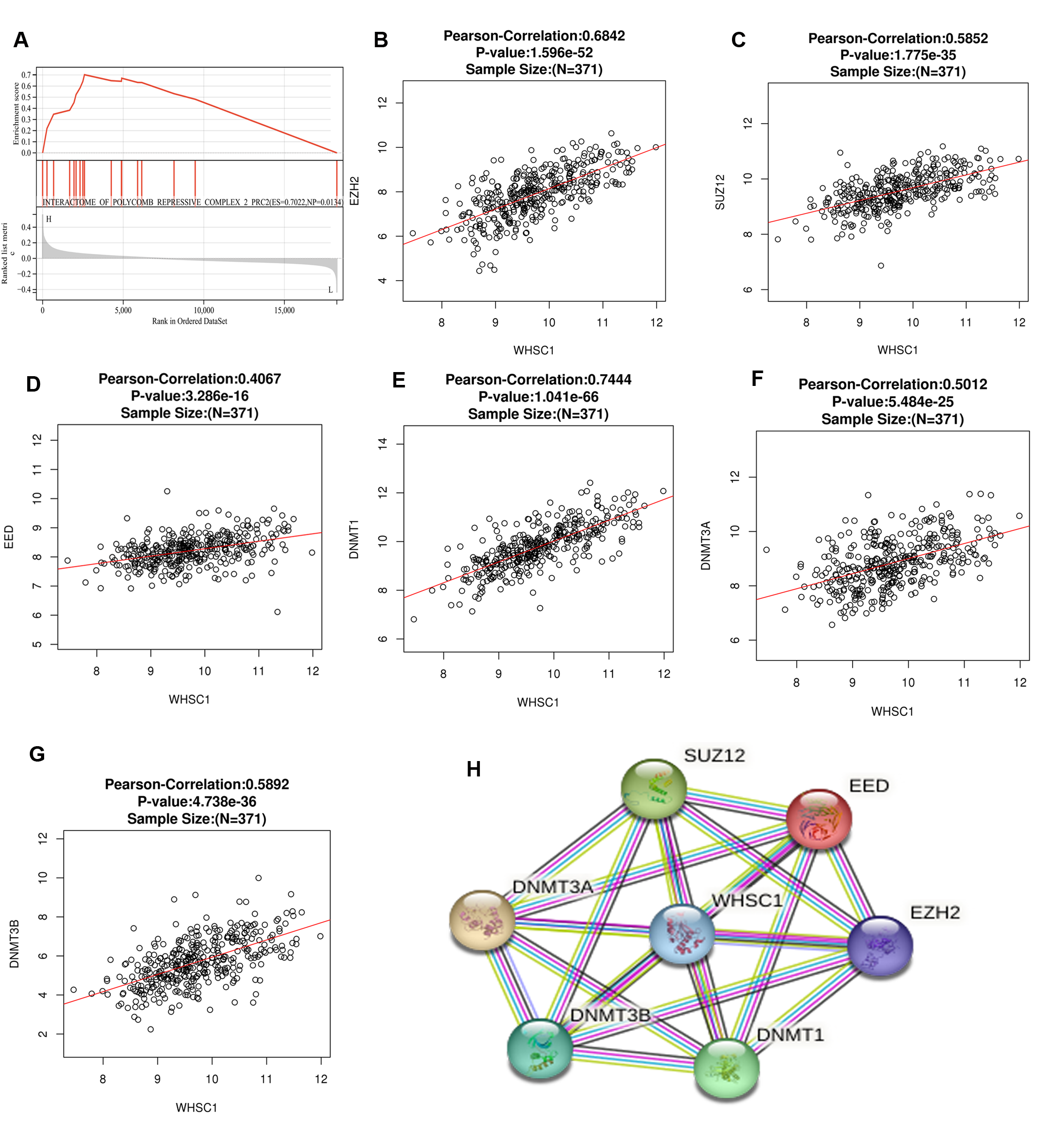

Supplement: Supplementary file 3 — Figure S3. [file JCMM-27-1436-s005.tif]

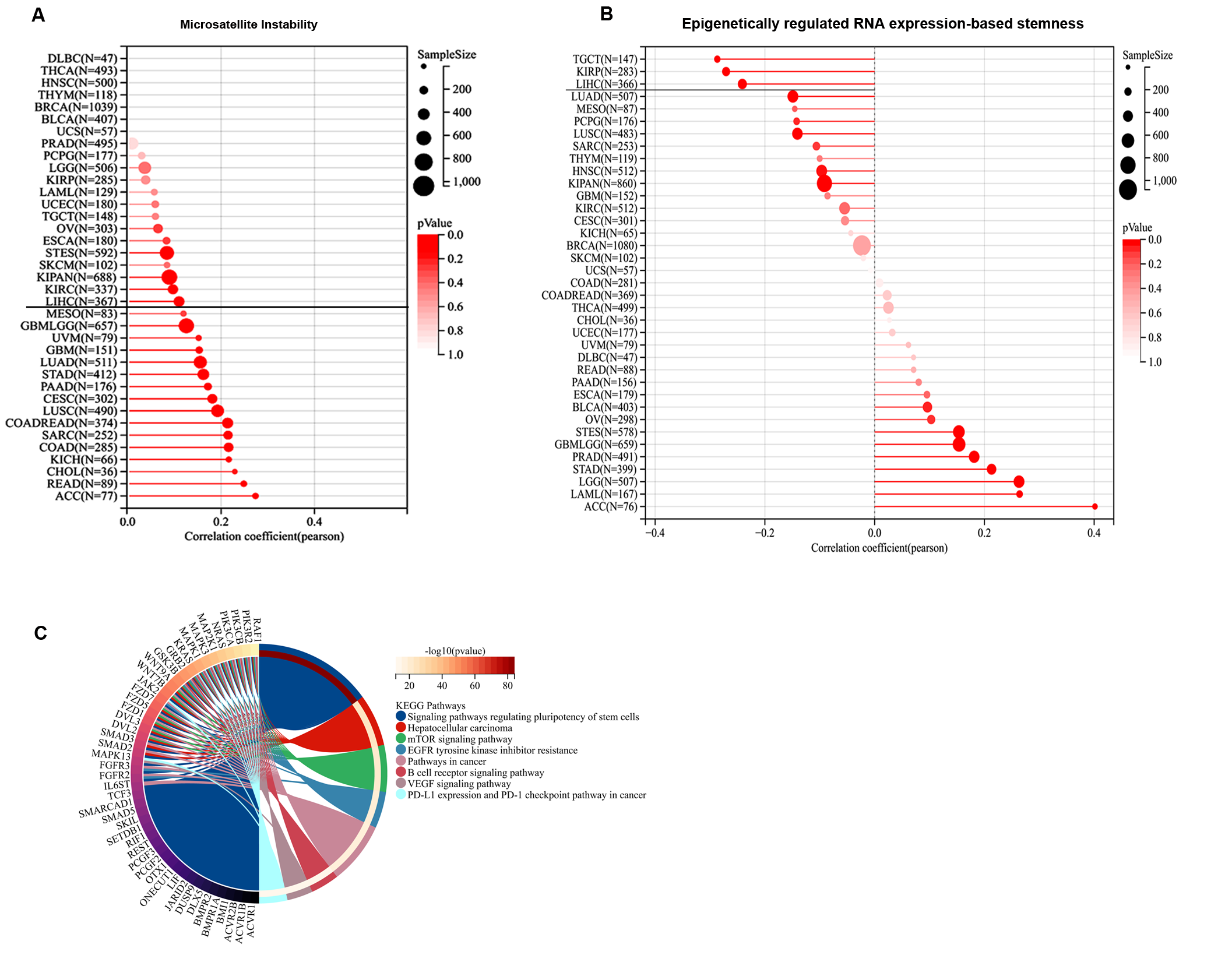

Supplement: Supplementary file 4 — Figure S4. [file JCMM-27-1436-s006.tif]
