# Supplementary material for: WHSC1 is involved in DNA damage, cellular senescence and immune response in hepatocellular carcinoma progression
Source: J Cell Mol Med. 2023 Apr 18;27(10):1436–41. doi: 10.1111/jcmm.17743 (PMC10183708; doi:10.1111/jcmm.17743)
Supplement: Supplementary file 5 — Table S1. [file JCMM-27-1436-s001.docx]

| Serial number | Function pathway |
| --- | --- |
| GO:0000018 | regulation of DNA recombination |
| [GO:0000726](http://www.ebi.ac.uk/QuickGO/GTerm?id=GO:0000726) | non-recombinational repair |
| [GO:0001501](http://www.ebi.ac.uk/QuickGO/GTerm?id=GO:0001501) | skeletal system development |
| [GO:0002200](http://www.ebi.ac.uk/QuickGO/GTerm?id=GO:0002200) | somatic diversification of immune receptors |
| [GO:0002204](http://www.ebi.ac.uk/QuickGO/GTerm?id=GO:0002204) | somatic recombination of immunoglobulin genes involved in immune response |
| [GO:0002208](http://www.ebi.ac.uk/QuickGO/GTerm?id=GO:0002208) | somatic diversification of immunoglobulins involved in immune response |
| [GO:0002250](http://www.ebi.ac.uk/QuickGO/GTerm?id=GO:0002250) | adaptive immune response |
| [GO:0002263](http://www.ebi.ac.uk/QuickGO/GTerm?id=GO:0002263) | cell activation involved in immune response |
| [GO:0002285](http://www.ebi.ac.uk/QuickGO/GTerm?id=GO:0002285) | lymphocyte activation involved in immune response |
| [GO:0002312](http://www.ebi.ac.uk/QuickGO/GTerm?id=GO:0002312) | B cell activation involved in immune response |
| [GO:0002366](http://www.ebi.ac.uk/QuickGO/GTerm?id=GO:0002366) | leukocyte activation involved in immune response |
| [GO:0002377](http://www.ebi.ac.uk/QuickGO/GTerm?id=GO:0002377) | immunoglobulin production |
| [GO:0002381](http://www.ebi.ac.uk/QuickGO/GTerm?id=GO:0002381) | immunoglobulin production involved in immunoglobulin mediated immune response |
| [GO:0002440](http://www.ebi.ac.uk/QuickGO/GTerm?id=GO:0002440) | production of molecular mediator of immune response |
| [GO:0002443](http://www.ebi.ac.uk/QuickGO/GTerm?id=GO:0002443) | leukocyte mediated immunity |

GO enrichment of WHSC1 in immune response pathways
