# Supplementary material for: WHSC1 is involved in DNA damage, cellular senescence and immune response in hepatocellular carcinoma progression
Source: J Cell Mol Med. 2023 Apr 18;27(10):1436–41. doi: 10.1111/jcmm.17743 (PMC10183708; doi:10.1111/jcmm.17743)
Supplement: Supplementary file 6 — AppendixS1. [file JCMM-27-1436-s004.docx]

**Figure Legends**

Figure S1. Gene expression of WHSC1 in different normal tissues.

Figure S2. Epigenetic modification analysis of WHSC1 in LIHC. (A) The correlations of WHSC1 and RNA modification related genes, including m1A, m5C and m6C. (B) The distribution of DNA methylation at the WHSC1 locus in LIHC. (C) The DNA methylation level of WHSC1 premotor in LIHC. (D, E) The most significantly associated methylation site of WHSC1 with prognosis.

Figure S3. WHSC1 was significantly associated with H3K27me3 and DNA methylation related genes. (A) WHSC1 co-expressed genes was associated with PCR2 complex. (B-D) The expression of WHSC1 was significantly correlated with PCR2 complex related enzymes, including EZH2 (B), SUZ12 (C), EED (D). (E-G) The correlations of WHSC1 and DNA methylation enzymes, DNMT1, DNMT3A and DNMT3B. (H) The protein interaction network of WHSC1 and H3K27me3 and DNA methylation related genes.

Figure S4. The relationships of WHSC1 and MIS or stemness in LIHC. (A) The expression of WHSC1 was associated with the microsatellite instability in several cancers, including LIHC. (B) The epigenetically regulated RNA expression of WHSC1 was correlated with several cancers. It is dramatically negatively associated with LIHC. (C) KEGG pathways of WHSC1-related and stemness regulated genes in LIHC.
